# The Zebrafish miR-183 Family Regulates Endoderm Convergence and Heart Development via S1Pr2 Signaling Pathway

**DOI:** 10.3390/biom15101434

**Published:** 2025-10-10

**Authors:** Ting Zeng, Ling Liu, Jinrui Lv, Hao Xie, Qingying Shi, Guifang Tao, Xiaoying Zheng, Lin Zhu, Lei Xiong, Huaping Xie

**Affiliations:** 1 Hunan International Joint Laboratory of Animal Intestinal Ecology and Health, Laboratory of Animal Nutrition and Human Health, College of Life Sciences, Hunan Normal University, Changsha 410081, China; tingz@hunnu.edu.cn (T.Z.); 202120142637@hunnu.edu.cn (L.L.); ljr0122@hunnu.edu.cn (J.L.); haox@hunnu.edu.cn (H.X.); 202370142925@hunnu.edu.cn (Q.S.); 202320142756@hunnu.edu.cn (G.T.); 202370142927@hunnu.edu.cn (X.Z.); 202420142862@hunnu.edu.cn (L.Z.); 2 School of Medical Information and Engineering, Hunan University of Medicine, Huaihua 418000, China

**Keywords:** zebrafish, endoderm, cardiac precursor cells, cardia bifida, S1Pr2

## Abstract

MicroRNA (miRNA), as a key post-transcriptional regulatory factor, plays a crucial role in embryonic development. The coordination of endoderm cell convergence and cardiac precursor cell (CPC) migration is critical for cardiac tube fusion. Defects in endoderm can impair the normal migration of CPCs towards the midline, leading to cardia bifida. Although the role of the microRNA-183 family (miR-183, miR-96 and miR-182) in cardiovascular diseases has been reported, the mechanism by which they regulate early heart development remains unclear. In this study, we used zebrafish as a model to elucidate the roles of the microRNA-183 family in early heart development. miRNA mimics were injected into Tg (*cmlc2*: eGFP) and Tg (*sox17*: eGFP) transgenic embryos to overexpress the miR-183 family. The results showed that, at 36 hpf, single or co-injection of miR-183/96/182 mimics caused defects in endoderm convergence, with a hole in the endoderm, and a significant down-regulation of the endoderm marker gene *sox32*. Additionally, embryos with single or co-injection of miR-183/96/182 mimics exhibited cardia bifida and tail blisters, with significantly down-regulated expression levels of genes related to heart development, including *cmlc2*, *vmhc*, *amhc*, *nppa*, *gata4*, *gata5*, *nkx2.5*, *bmp2b*, and *bmp4*. The phenotype caused by overexpression of the miR-183 family is highly consistent with loss of the sphingosine 1-phosphate receptor S1Pr2. Bioinformatics analysis result found that miR-183 can bind to 3′-UTR of the *s1pr2* to regulate its expression; overexpression of miR-183 led to a significant decrease in the expression of the *s1pr2* gene. Dual luciferase assay results suggest that *s1pr2* is a bona fide target of miR-183. In summary, the miR-183 family regulates endoderm convergence and cardiac precursor cell migration via the S1Pr2 signaling pathway. This study reveals that the miR-183 family is a key regulatory factor in endoderm convergence and cardiac precursor cell migration during the early zebrafish development, elucidating the molecular mechanisms underlying early cardiac precursor cell and endoderm cell movement.

## 1. Introduction

As one of the three primary germ layers formed earliest during embryonic development, the endoderm serves as the foundation for the development of the majority of the body’s organs. During organogenesis, this germ layer differentiates to establish the fundamental structures of the digestive tract and respiratory systems, giving rise to vital organs such as the hepatobiliary system, pancreas, and intestines [1]. The endoderm’s capacity for multi-system and cross-organ differentiation underscores its central role in maintaining the body’s essential physiological functions. Abnormalities in early development can lead to severe congenital anomalies, including cardiac structural defects, congenital diaphragmatic hernia, reproductive system malformations, and embryonic lethality. Therefore, the proper migration of endodermal cells during early development is critical for ensuring normal physiological function [2].

The endoderm lies adjacent to the cardiac mesoderm and is critical for migration of cardiac precursor cells (CPCs). During embryogenesis, zebrafish CPCs originating from the anterior lateral plate mesoderm and then migrate towards to the midline, forming the primitive heart tube [3]. Zebrafish endoderm loss-of-function mutants (*cas*/*sox32*, *sox17*, *oep*, *fau*/*gata5*, and *bon*), as well as mouse endoderm-deficient mutants (*foxp4* and *gata4*), exhibit cardia bifida phenotype, characterized by two laterally positioned hearts (cardiac tubes fail to fuse properly in the midline) [4,5,6]. Similarly, endoderm disruption in chick embryos disrupts cardiomyocyte migration [7].

The endoderm regulates heart formation by expressing growth factors essential for cardiac specification and differentiation [8]. In zebrafish, sphingosine-1-phosphate (S1P) signaling regulates cardiomyocyte migration via its cognate G protein-coupled receptor, S1pr2 [9]. The s1pr2 (*mil*) mutant exhibits cardia bifida, demonstrating that the S1P/S1pr2 signaling pathway is essential for CPC migration [10]. S1pr2 activates RhoGEF by coupling with Gα_13_ protein to regulate endoderm convergence and coordinate cardiomyocyte migration [11]. Disruption of S1pr2/Gα_13_ signaling leads to defects in endodermal convergence, impairing cardiomyocyte migration [12,13]. Further studies show that the S1pr2/Gα_13_/Rho pathway activates cell surface integrins and promotes the assembly of fibronectin, thereby providing the correct migratory direction for endodermal cells and enabling the collective migration of cardiac precursor cells towards the midline [14].

MicroRNA-183 (miR-183) is a small non-coding RNA regulating genes post-transcriptionally. It belongs to the miR-183 family, a highly conserved miRNA cluster. This family also includes miR-96 and miR-182 [15]. Members of this family are located within a 5 kb region on human chromosome 7q32.2, are transcribed in the same direction (from telomere to centromere) and have similar biological functions in signaling pathways [16]. The miR-183 family is expressed in the eye and inner ear and plays crucial roles in the development of retina and inner ear hair cells [17,18]. Furthermore, studies have shown that the miR-183 family is highly expressed in a variety of cancers, with a close association with the occurrence and progression of breast cancer, lung cancer, colorectal cancer, and hepatocellular carcinoma [19,20]. These findings suggest that the miR-183 family could be a potential biomarker for cancer diagnosis and prognosis [21,22]. As an oncogene, miR-183 promotes tumor growth and metastasis [23,24] and participates in various cellular processes such as tumor cell proliferation, apoptosis, and metabolism by regulating the expression of its target genes [25,26]. In recent years, the functions of the miR-183 family in cardiovascular diseases have been revealed [27,28,29,30]. Double knockout of miR-96/miR-183 in mice promotes neovascularization [29]. miR-183 expression correlates with heart failure in adults with transposition of the great arteries and systemic right ventricular dysfunction [31]. miR-183 protects rats against myocardial ischemia/reperfusion injury by targeting *VDAC1* [30]. Furthermore, miR-182 has been identified as a downstream effector of the key cardiac development transcription factor *tbx5*, which participates in the regulation of cardiac morphogenesis and electrophysiological activity in zebrafish [32]. However, the functions of the miR-183 family in early heart development remain unclear.

This study aimed to elucidate the functions of the miR-183 family in early heart development of zebrafish. We found that overexpression of the miR-183 family caused embryonic phenotypes such as endodermal convergence defects (holes), cardia bifida, and tail blisters. Additionally, bioinformatics analysis uncovered *s1pr2* as a potential target gene of the miR-183 family, and dual luciferase assays confirmed a genetic interaction between miR-183 and *s1pr2*. In summary, this study revealed that the miR-183 family regulates the S1Pr2 signaling pathway, affecting endoderm convergence, thereby coordinating the migration of cardiac precursor cells and heart tube fusion. These findings provide insight into the molecular mechanism governing the movement of early cardiac precursor and endoderm cells.

## 2. Materials and Methods

### 2.1. Zebrafish Breeding and Ethical Considerations

Tuebingen (TU) line, transgenic line Tg (*cmlc2*: eGFP) and Tg (*sox17*: eGFP) zebrafish were bred and maintained in our laboratory under the following conditions: a water temperature of 28 °C, pH 6.5–7.5, salinity of 450–500 μs/cm, and a 14 h/10 h light and dark cycle. Fish were crossed weekly; the embryos were cultured in E3 solution at 28.5 °C. All animal experiments were approved by the Biomedical Research Ethics Committee of Hunan Normal University (approval No. 2021/196, 1 January 2022).

### 2.2. miRNA Mimic Microinjection

Mature sequences for miR-183, miR-96, and miR-182 genes were identified using the miRBase database, and corresponding mimics were synthesized by Gema Biotechnology Co., Ltd. (Olivos, Argentina), and injected into zebrafish embryos at the 1-cell stage. The injection concentrations were as follows: miR-183 mimics (3.5 nM), miR-96 mimics (4 nM), and miR-182 mimics (4 nM). The injection concentrations in the miRNA mixture experiment were as follows: miR-183 mimics (0.95 nM), miR-96 mimics (1.19 nM), and miR-182 mimics (1.19 nM).

### 2.3. Whole-Mount In Situ Hybridization (WISH)

Zebrafish embryos were fixed overnight at 4 °C in 4% paraformaldehyde (PFA), then washed twice with PBST, digested with a 10 mg/mL proteinase K solution, refixed at room temperature with 4% PFA for 20 min, and rinsed twice with PBST. They were then hybridized overnight at 68 °C with a DIG-labeled antisense RNA probe. On the second day, after washing with 50% formamide/2X SSCT, 2X SSCT, and 0.2X SSCT solutions, the embryos were incubated overnight with anti-DIG antibody. After washing three times with MABT solution, with 25-min intervals, the embryos were stained with BCIP-NBT solution and imaged using a Leica stereomicroscope (Leica Microsystems, Wetzlar, Germany). Digoxigenin-UTP-labeled antisense RNA probes for the *cmlc2*, *vmhc*, *gata4*, *nppa*, and *sox32* genes were synthesized using the T7 in vitro transcription kit (Thermo Fisher Scientific, Waltham, MA, USA) via in vitro transcription. The primer sequences for these genes are listed in Table S1.

### 2.4. Real-Time Quantitative PCR (RT-qPCR)

Fifty control and miR-183-overexpressing embryos at 42 hpf were collected in triplicate, and rapidly frozen in liquid nitrogen and stored at −80 °C. Total RNA was extracted from the embryos using TRIZOL (TaKaRa, Dalian, China) reagent according to the manufacturer’s instructions. Total RNA (1 μg) was reverse transcribed into cDNA using reverse transcriptase (TaKaRa, Dalian, China) and oligonucleotide primers. Quantitative PCR was performed using 2×SYBR Green Master qPCR Mix (Vazyme, Nanjing, China) to detect the expression of related genes. The qPCR primers used in this study are listed in Table S2. Relative expression levels of the tested mRNAs were determined using *18s* as an internal reference and the comparative Ct (2^−ΔΔCt^) method.

### 2.5. Dual Luciferase Reporter Assay

A dual luciferase reporter assay was performed in HEK293T cells. 293T cells were seeded in a 24-well plate and cultured in serum-reduced medium until the cell confluence reached 60–70%. After starvation for 1 h, cells were co-transfected with Lipofectamine 2000 (Invitrogen, Carlsbad, CA, USA) containing miR-NC (100 nM) or miR-183 mimics (100 nM) along with the reporter gene containing the *s1pr2* wild-type (WT) or mutant (MUT) 3′-UTR sequence. The transfected cells were cultured at 37 °C and 5% CO_2_ for 6 h, then the medium was replaced with complete medium and the cells were cultured for another 24 h. The luciferase activity was measured using a dual luciferase reporter gene assay kit (Promega, Madison, WI, USA) according to the manufacturer’s instructions.

### 2.6. Statistical Analysis

All experimental data were obtained from at least three independent experiments. Significant differences between different types of embryos were calculated using *t*-tests; *p* < 0.05 was considered statistically significant.

## 3. Results

### 3.1. The miR-183 Family Is Highly Conserved in Vertebrates

**Figure 1:**
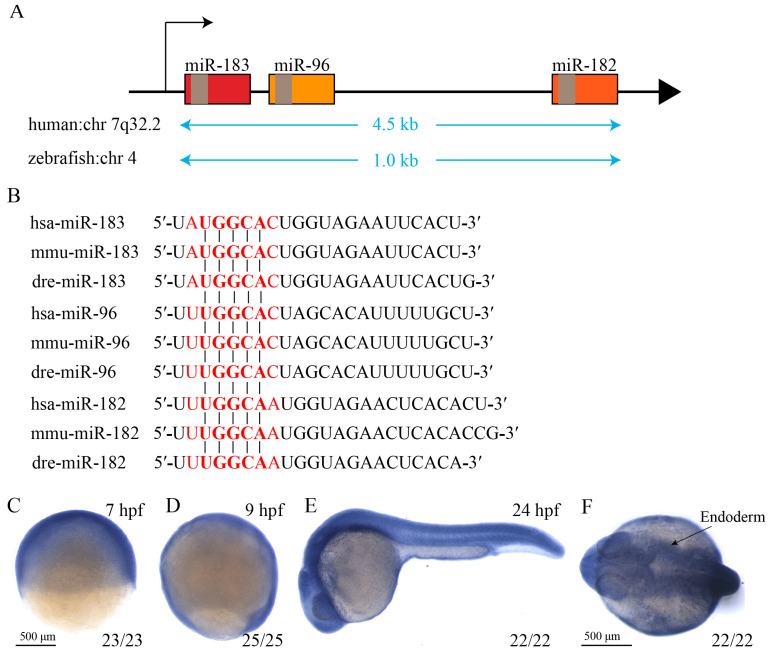
The miR-183 family is highly conserved. (**A**) Schematic diagram of the genomic localization of the miR-183 gene cluster on human chromosome 7 long arm 32.2 (7q32.2) and zebrafish chromosome 4. (**B**) Mature sequence alignment of miR-183, miR-96, and miR-182 sequences in humans (hsa-), mice (mmu-), and zebrafish (dre-). The seed sequence (nucleotides 2–8) is highlighted in red. (**C**,**D**) miR-183 was ubiquitously expressed at the 7 hpf to 9 hpf stage. (**E**,**F**) miR-183 was expressed in the endoderm at 24 hpf, lateral view (**E**), dorsal view (**F**). The numbers in the lower right corner indicate the number of embryos.

The miR-183 family consists of three members: miRNA-183, miRNA-96, and miRNA-182. In 2002, miR-96 was first identified in human cancer cell lines [33]. Subsequently, in 2003, researchers discovered miR-182 and miR-183 by comparing genomic RNA differences among humans, rats, and Japanese red porpoises [34]. In the human genome, the miR-183 family is tightly linked and located in the intergenic region of chromosome 7q32.2. In the zebrafish genome, this family is located on chromosome 4 (Figure 1A). miRNAs primarily exert post-transcriptional regulatory effects by binding to the 3′-untranslated region (3′-UTR) of target gene mRNAs via their highly conserved seed sequences. We aligned the mature nucleotide sequences of the miR-183 family from humans, mice, and zebrafish. The results showed 100% identity in the seed sequences of miR-183, miR-96, and miR-182 across these three species (Figure 1B), indicating high evolutionary conservation of the core targeting function within this miRNA family. Although the family members exhibit high overall sequence similarity, subtle differences exist in their seed sequences (Figure 1B), which may lead to the recognition of different target RNAs and mediate distinct regulatory networks. Next, we employed whole-mount in situ hybridization (WISH) using a miR-183 anti-sense RNA probe to examine the spatiotemporal expression pattern of miR-183 during embryonic development. The WISH results demonstrated that miR-183 was ubiquitously expressed from 7 to 9 hpf (Figure 1C,D) and expressed in the endoderm at 24 hpf (Figure 1E,F). This finding suggests that miR-183 plays a role in the early development of zebrafish.

### 3.2. Overexpression of miR-183 Affects Early Heart Development

**Figure 2:**
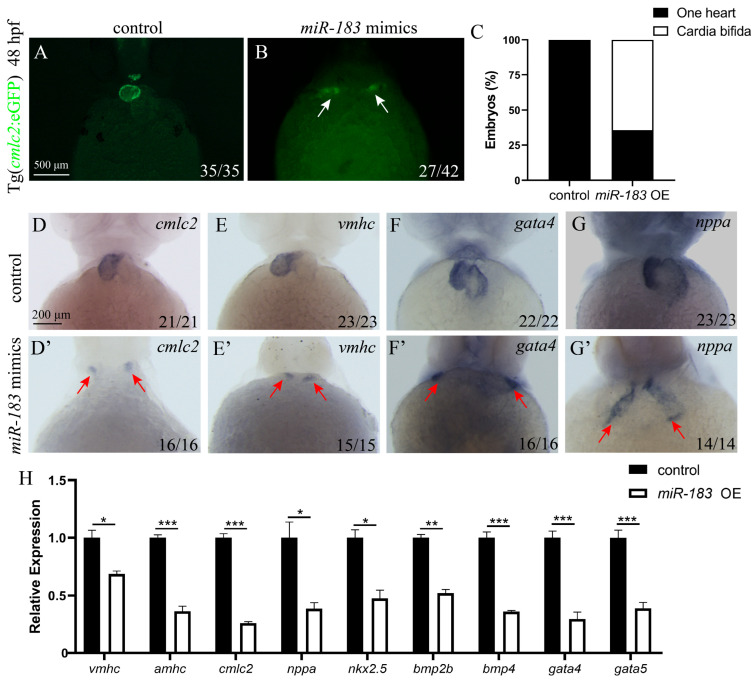
Overexpression of miR-183 causes cardia bifida in zebrafish. (**A**,**B**) Fluorescence images of the heart of 48 hpf Tg (*cmlc2*: eGFP) transgenic zebrafish embryos (ventral view). (**A**) Control embryos showing a normal fused tubular heart, (**B**) Embryos injected with miR-183 mimic exhibit cardia bifida (white arrow heads indicate two separate cardiac primordia). (**C**) Quantification of cardia bifida phenotype in miR-183 OE embryos. (**D**–**G**) Whole-mount in situ hybridization detection of *cmlc2* (**D**,**D’**), *vmhc* (**E**,**E’**), *gata4* (**F**,**F’**), and *nppa* (**G**,**G’**) expression in 48 hpf control and miR-183 OE embryos (ventral view; red arrow heads indicate two separate cardiac primordia). (**H**) RT-qPCR analysis of the mRNA expression levels of cardiac developmental genes at 42 hpf miR-183 OE embryos compared to wild-type. The numbers in the lower right corner indicate the number of embryos. * *p* < 0.05, ** *p* < 0.01, *** *p* < 0.001.

To investigate the role of the miR-183 family in zebrafish early development, we performed a miR-183 overexpression assay (miR-183 OE). A total of 1 nL of miR-183 mimic (3.5 nM) was microinjected into 1-cell stage embryos of the *cmlc2*: eGFP transgenic line, which expresses green fluorescence specifically in cardiomyocytes, and its effects on embryonic development were observed. At 48 h post-fertilization (hpf), control embryos showed normal migration of cardiac precursor cells to the midline, where they fused to form a single heart tube. In contrast, miR-183 OE embryos exhibited cardia bifida (double hearts) (Figure 2A–C). These results indicate that overexpression of miR-183 disrupted cardiomyocyte migration in zebrafish. To further investigate whether miR-183 overexpression affects early heart development, we detected the expression levels of heart development marker genes by WISH and RT-qPCR. WISH results showed that, compared with the control, the expression of the cardio myosin marker *cmlc2*, the ventricular marker *vmhc*, and the ventricular development genes *gata4*, and *nppa* was ectopic in miR-183 OE embryos, exhibiting a phenotype of two independent hearts (Figure 2D–G). RT-qPCR data revealed that the mRNA expression levels of multiple key genes were significantly down-regulated in miR-183 OE embryos, compared with the control, including *cmlc2*, *vmhc*, the atrial marker *amhc*, the ventricular development genes *nppa*, *gata4*, and *gata5*, the cardiac mesoderm marker *nkx2.5*, and the cardiac development-related transcription factors *bmp2b* and *bmp4* (Figure 2H). These results further demonstrate that miR-183 overexpression caused cardiac precursor cells to fail to migrate correctly to the midline and fuse into a single heart tube.

### 3.3. miR-183 Overexpression Affects Endoderm Convergence

**Figure 3:**
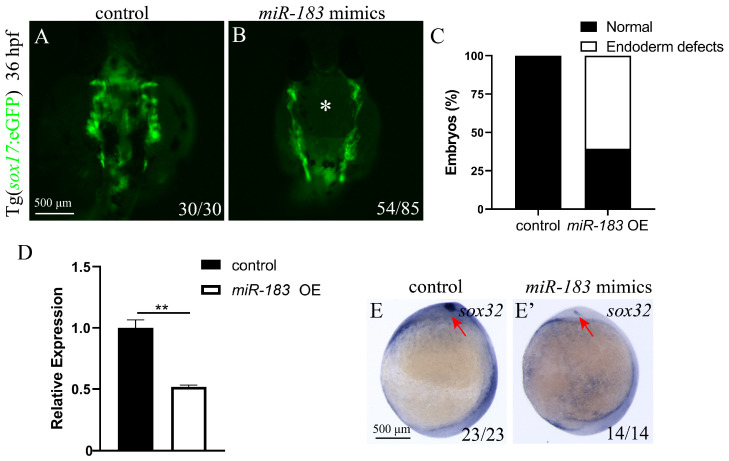
Overexpression of miR-183 disrupts endoderm convergence movement. (**A**,**B**) Fluorescence image of the endoderm of transgenic zebrafish embryos with Tg (*sox17*: GFP) at 36 hpf (ventral view). (**A**) Control embryos show normal endoderm cell convergence. (**B**) miR-183 OE embryos exhibit endodermal defects with holes in the central region of the tissue (indicated by white asterisk). Scale bar: 500 μm. (**C**) Quantification of endodermal convergence defects in miR-183 OE embryos. (**D**) *sox32* mRNA expression levels were analyzed by RT-qPCR in wild-type and miR-183 OE embryos at 36 hpf. (**E**,**E’**) WISH detecting *sox32* expression in control and miR-183 OE embryos at 9 hpf (ventral view; red arrow heads indicate the kupffer’s vesicle). The numbers in the lower right corner indicate the number of embryos. ** *p* < 0.01.

Previous studies have shown that the endoderm is crucial for the migration of cardiac precursor cells, and its dysfunction disrupts cardiomyocyte migration, leading to cardia bifida [35]. To determine whether miR-183 overexpression-induced cardia bifida stems from impaired endodermal development, we injected miR-183 mimics into *sox17*: eGFP embryos at the 1-cell stage and observed endoderm development at 36 hpf. Compared with the control (Figure 3A), 65% of miR-183 OE embryos showed a hole in the endoderm (Figure 3B,C). Next, we detected the expression of the early endoderm development marker *sox32* by RT-qPCR and WISH. RT-qPCR results showed that *sox32* expression was significantly down-regulated (Figure 3D). WISH results also showed a reduced *sox32* expression in miR-183 OE embryos compared with the control (Figure 3E,E’). These results suggest that miR-183 is required for endoderm convergence.

### 3.4. Overexpression of miR-96 and miR-182 Leads to Cardia Bifida

**Figure 4:**
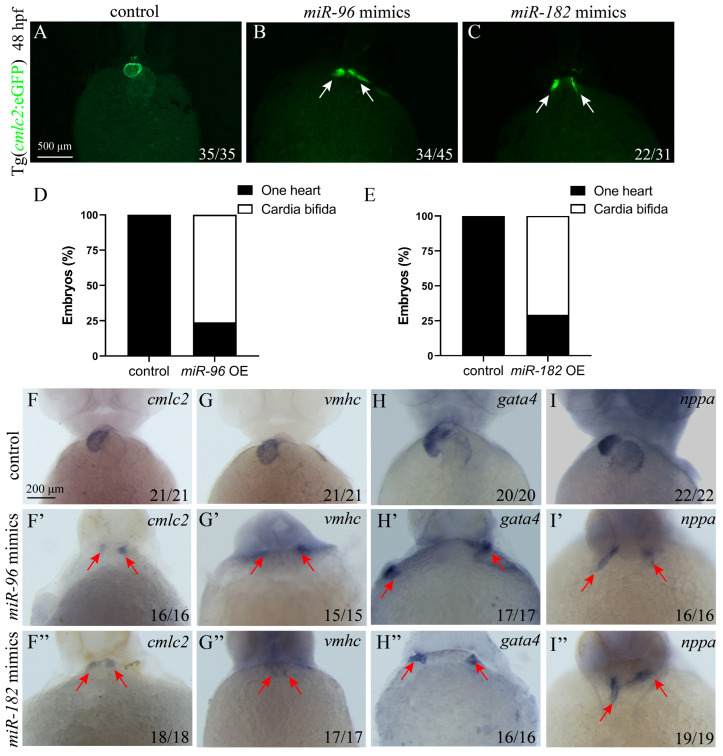
miR-96/182 overexpression leads to cardia bifida. (**A**–**C**) 48 hpf Tg (*cmlc2*: eGFP) transgenic zebrafish embryo cardiac fluorescence image (ventral view). (**A**) Control embryos show a normally fused heart tube (green). Embryos of miR-96 OE (**B**) and miR-182 OE (**C**) exhibit cardia bifida phenotypes (white arrow heads indicate two separated cardiac primordia). (**D**,**E**) Quantification of cardia bifida in miR-96 OE and miR-182 OE embryos. (**F**–**I**) WISH detected the expressions of *cmlc2* (**F**,**F’**,**F”**), *vmhc* (**G**,**G’**,**G”**), *gata4* (**H**,**H’**,**H”**), and *nppa* (**I**,**I’**,**I”**) in the control, miR-96 OE, and miR-182 OE embryos at 48 hpf (red arrow heads indicate two separated cardiac primordia). The numbers in the lower right corner indicate the number of embryos.

The above studies indicate that miR-183 overexpression causes cardia bifida by interfering with endoderm and cardiac precursor cell migration. To explore whether other miR-183 family members (miR-96, miR-182) similarly affect cardiac precursor cells migration, we injected miR-96 (4 nM) and miR-182 mimics (4 nM) into 1-cell stage *cmlc2*: eGFP zebrafish embryos and detected at 48 hpf. The results showed that both miR-96 OE and miR-182 OE embryos exhibited cardia bifida, in contrast to the control (Figure 4A–E). These results indicate a conserved function of the miR-183 family in regulating the early fusion of the zebrafish heart tube. Next, to further investigate whether overexpression of miR-96 and miR-182 affects the differentiation of cardiomyocytes, we detected the expression of cardiac development markers through WISH. WISH results revealed reduced expression and ectopic distribution of *cmlc2*, *vmhc*, *gata4*, and *nppa* in miR-96 OE and miR-182 OE embryos compared with the control (Figure 4F–I).

### 3.5. miR-96 and miR-182 Overexpression Resulted in Endodermal Defects

**Figure 5:**
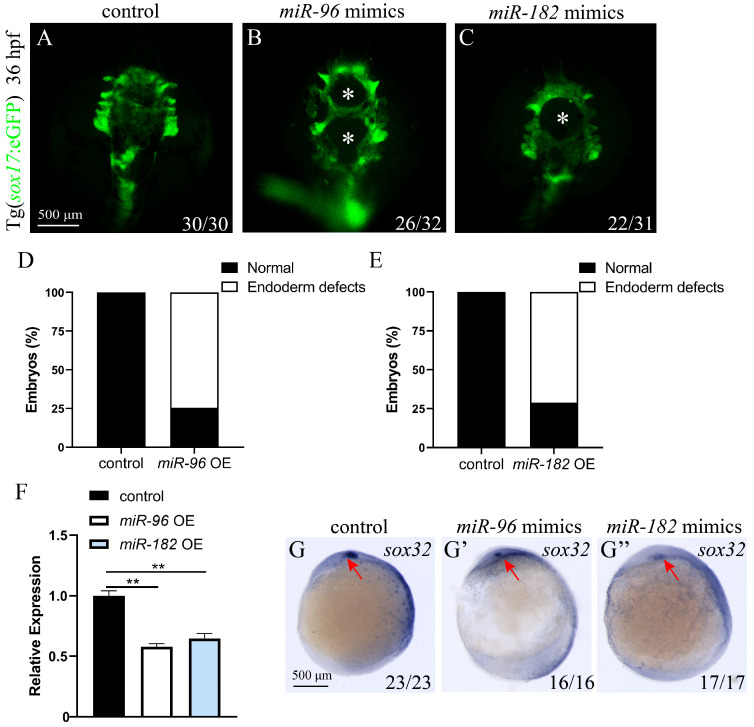
Overexpression of miR-96 and miR-182 leads to defects in endoderm cell convergence. (**A**–**C**) Fluorescence image of the endoderm of 36 hpf Tg (*sox17*: eGFP) transgenic zebrafish embryo (ventral view). Control embryos display normal endoderm cell convergence (**A**); both miR-96 OE (**B**) and miR-182 OE (**C**) embryos exhibit endodermal defects with holes in the central (white asterisks). (**D**,**E**) Quantification of endodermal defects in miR-96 OE (**D**) and miR-182 OE (**E**) embryos. (**F**) *sox32* mRNA expression levels were analyzed by RT-qPCR in control, miR-96 OE, and miR-182 OE embryos at 36 hpf. (**G**–**G”**) WISH detected the expression of *sox32* in control (**G**), miR-96 OE (**G’**), and miR-182 OE (**G”**) embryos at 9 hpf (ventral view; red arrow heads indicate the kupffer’s vesicle). The numbers in the lower right corner indicate the number of embryos. ** *p* < 0.01.

Members of the miR-183 family have high sequence conservation and similar biological functions [18]. Moreover, the aforementioned studies have shown that overexpression of miR-183, miR-96, and miR-182 can all lead to abnormal migration of cardiac precursor cells. Therefore, to investigate whether overexpression of miR-96 and miR-182 similarly affects endoderm cell migration, we injected miR-96 mimics (4 nM) and miR-182 mimics (4 nM) into *sox17*: eGFP zebrafish embryos at the 1-cell stage and observed the development of the endoderm at 36 hpf. The results were consistent with the miR-183 overexpression phenotype, both miR-96 OE and miR-182 OE embryos showed endoderm defects with holes in the central region (Figure 5A–E). Next, we detected the expression of the early endoderm developmental marker *sox32* using RT-qPCR and WISH. RT-qPCR results showed that compared with the control, the expression of *sox32* was significantly down-regulated in miR-96 OE and miR-182 OE embryos (Figure 5F). WISH results also showed decreased *sox32* expression in miR-96 OE and miR-182 OE embryos compared with the control (Figure 5G–G”). These data indicate that miR-96 and miR-182 regulate endoderm cell convergence.

### 3.6. Co-Expression of the miR-183 Family Affects Endoderm Convergence and Cardiac Precursor Cell Migration

**Figure 6:**
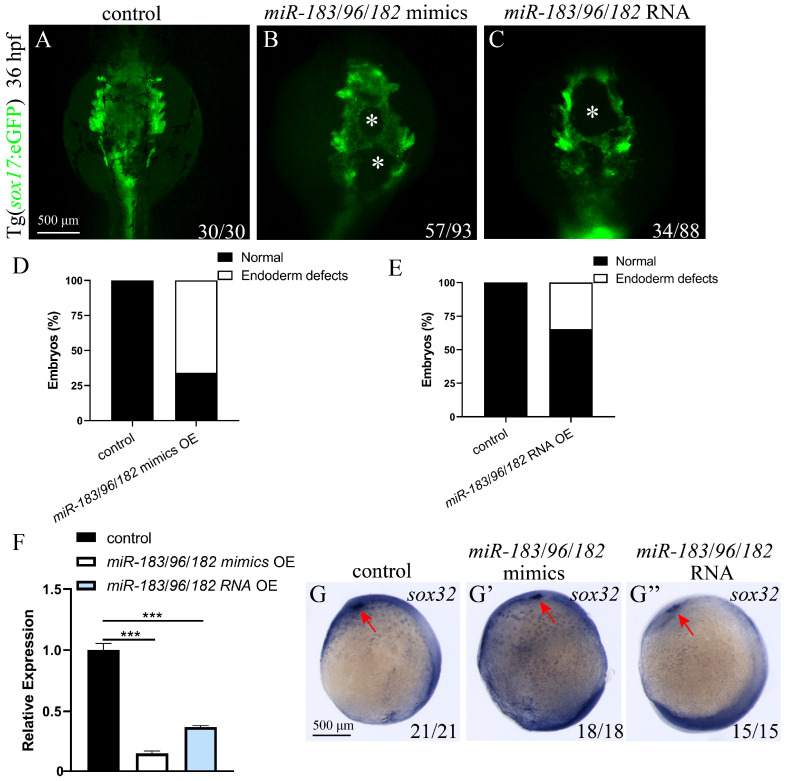
Co-expression of the miR-183 family leads to abnormal migration of endodermal cells. (**A**–**C**) Fluorescence image of the endoderm of 36 hpf Tg (*sox17*: eGFP) transgenic zebrafish embryo (ventral view). Control embryos show normal convergence of endoderm cells at the midline (**A**); Embryos of miR-183/96/182 mimics OE (**B**) and miR-183/96/182 capped RNA OE (**C**) both presented with holes in the central region of the endoderm tissue (indicated by white asterisks). (**D**,**E**) Quantification of endodermal defect phenotypes in miR-183/96/182 mimics OE (**D**) and miR-183/96/182 capped RNA OE (**E**) embryos. (F) The mRNA expression levels of the *sox32* gene in the control, miR-183/96/182 mimics OE and miR-183/96/182 capped RNA OE embryos were analyzed by RT-qPCR at 36 hpf. (**G**–**G”**) WISH was used to detect the expression of *sox32* in 9 hpf control (**G**), miR-183/96/182 mimics OE (**G’**), and miR-183/96/182 capped RNA OE (**G”**) embryos (ventral view; red arrow heads indicate the kupffer’s vesicle). The number at the bottom right indicates the number of embryos. *** *p* < 0.001.

**Figure 7:**
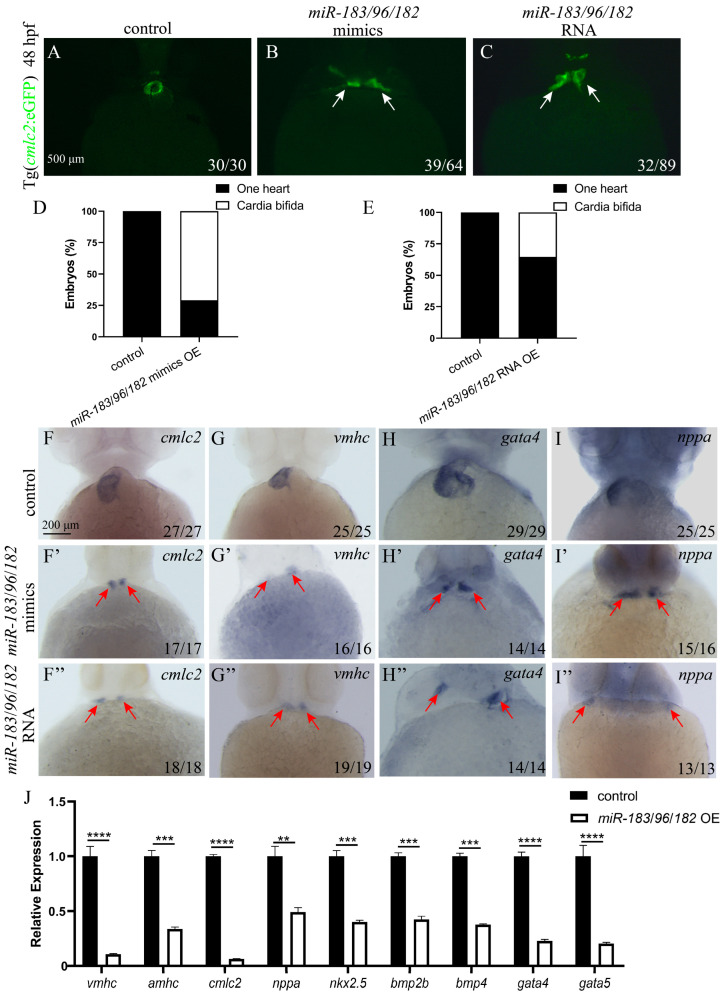
Co-expression of the miR-183 family leads to abnormal migration of myocardial precursor cells. (**A**–**C**) Fluorescence images of the hearts of Tg (*cmlc2*: eGFP) transgenic zebrafish embryos at 48 hpf (ventral view). Control embryos exhibit a normally fused heart tube (**A**) (green). Both miR-183/96/182 mimics OE (**B**) and miR-183/96/182 capped RNA OE (**C**) embryos exhibit cardia bifida (white arrow heads indicate separate cardiac primordia). (**D**,**E**) Quantification of cardia bifida in miR-183 cluster mimics OE (**D**) and miR-183/96/182 capped RNA OE (**E**) embryos. (**F**–**I**) WISH detected *cmlc2* (**F**,**F’**,**F”**), *vmhc* (**G**,**G’**,**G”**), *gata4* (**H**,**H’**,**H”**), and *nppa* (**I**,**I’**,**I”**) in embryos of the 48 hpf control, miR-183/96/182 mimics OE and miR-183/96/182 capped RNA OE (red arrow heads indicate separate cardiac primordia). (**J**) The mRNA expression levels of heart development-related genes in control, miR-183/96/182 mimics OE embryos were analyzed by RT-qPCR at 42 hpf. The number at the bottom right indicates the number of embryos. ** *p* < 0.01, *** *p* < 0.001, **** *p* <0.0001.

Previous results showed that miR-183 family members miR-183, miR-96, and miR-182 all affect endoderm and cardiomyocyte precursor cell migration. To further explore whether there is a synergistic effect or functional redundancy among family members, we co-expressed miR-183 family mimics. A mixture of miR-183, miR-96, and miR-182 mimics was injected into 1-cell stage *sox17*: eGFP or *cmlc2*: eGFP embryos, and endoderm and heart development was observed at 36 hpf and 48hpf. The results showed that, compared with the control, embryos with co-overexpression of the miR-183 family (miR-183/96/182 mimics) exhibited endodermal defects with holes in the central region (Figure 6A,B,D), as well as two hearts (Figure 7A,B,D). This phenotype is consistent with that observed upon single overexpression of miR-183 family members. We detected the expression of early heart and endoderm markers by WISH and RT-qPCR. WISH results confirmed that, compared with wild-type embryos, the expression of early endoderm developmental marker *sox32* was reduced in embryos co-expressing miR-183 family mimics (Figure 6G,G’), and *cmlc2*, *vmhc*, *gata4*, and *nppa* were mis-localized with decreased expression levels (Figure 7F–I). RT-qPCR results showed that the expression of *sox32* was significantly downregulated in co-expressing embryos compared with the control (Figure 6F). Similarly, the expression of early heart developmental markers *cmlc2*, *vmhc*, *amhc*, *nppa*, *gata4*, *gata5*, *nkx2.5*, *bmp2b*, and *bmp4* was significantly downregulated, compared with the control (Figure 7J). To exclude potential off-target effects of the mimics and validate the phenotype specificity, we synthesized the capped RNA of the miR-183-96-182 cluster in vitro and injected it into 1-cell stage *cmlc2*: eGFP or *sox17*: eGFP embryos. Endoderm and heart development was observed at 36 and 48 hpf. The results showed that embryos injected with capped RNA exhibited cardia bifida and endoderm hole phenotypes (Figure 6 and Figure 7), consistent with the results from miR-183 family mimics experiments. These results indicate that the miR-183 family regulates endodermal and cardiac precursor cells migration.

### 3.7. miR-183 Family Regulates Endodermal and Cardiac Precursor Cell Migration via the S1pr2 Signaling Pathway

**Figure 8:**
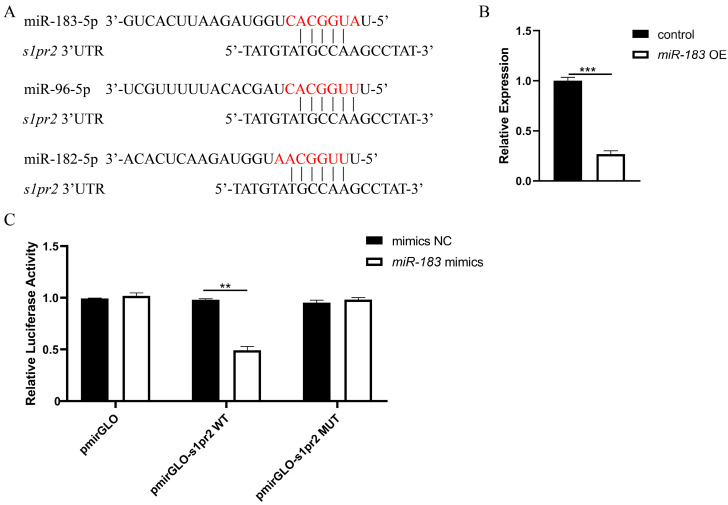
*s1pr2* is a target gene of the miR-183 family. (**A**) Binding sites between the miR-183 family and *s1pr2* based on the TargetScan database.The red character represents the seed sequence. (**B**) The mRNA expression levels of *s1pr2* in wild-type embryos and miR-183 OE embryos were analyzed by RT-qPCR at 42 hpf. (**C**) A dual-luciferase reporter assay was used to verify the binding of miR-183 and *s1pr2*. ** *p* < 0.01, *** *p* < 0.001.

Previous studies have shown that the abnormal function of S1pr2 leads to cardia bifida and endodermal holes [10]. In this study, overexpression of each member of the miR-183 family resulted in phenotypes consistent with S1Pr2 signaling pathway deficiency, suggesting that they may target and regulate this pathway. To verify this hypothesis, we used online target prediction algorithms TargetScan (http://www.targetscan.org/, accessed on 1 August 2023) and PicTar (http://www.pictar.org/, accessed on 2 August 2023) to identify potential targets of miR-183, and found that the seed sequences of miR-183 family members could bind to the 3’-UTR of the *s1pr2* gene, suggesting that *s1pr2* is a potential direct target gene of the miR-183 family (Figure 8A). RT-qPCR results showed that the mRNA expression level of *s1pr2* was significantly down-regulated in miR-183 OE embryos (Figure 8B). To further validate the genetic interaction between the miR-183 family and *s1pr2*, we cloned the wild-type (WT) 3′-UTR and mutant (MUT) 3′-UTR fragments of the *s1pr2* gene into a luciferase reporter vector (pmirGLO vector). Compared with the control, renilla luciferase activity was significantly reduced after co-transfection of the wild-type reporter with miR-183 mimic, whereas the mutant reporter showed no significant change in luciferase activity after co-transfection with miR-183 mimic (Figure 8C). This result indicates that miR-183 regulates *s1pr2* expression by binding to its 3′-UTR. Collectively, these results demonstrate that the miR-183 family regulates the migration of endodermal and cardiac precursor cells through the S1Pr2 signaling pathway.

## 4. Discussion

MicroRNAs (miRNAs) are crucial post-transcriptional regulators that play an important role in cell differentiation and fate determination during development [36]. However, the mechanisms underlying miRNA-mediated regulation of early embryonic development remain poorly understood. This study reveals the key functions and mechanisms of the miR-183 cluster (miR-183/96/182) in early heart development in zebrafish. Our research indicates that the miR-183 family targets the expression of the G protein-coupled receptor *s1pr2*, disrupting the sphingosine-1-phosphate (S1P) signaling pathway. This disruption impairs the convergence movement of endodermal cells and affects the migration of cardiac precursor cells towards the midline, leading to developmental defects such as endodermal holes and cardia bifida. These phenotypic characteristics are highly similar to the phenotype of *s1pr2* gene function loss [10]. Notably, we demonstrated a significant genetic interaction between miR-183 and *s1pr2*. In summary, our findings reveal a novel function for the miR-183 family in regulating endoderm and cardiac precursor cells migration by modulating *s1pr2* expression.

miRNAs play a crucial role in cell specialization, proliferation, and differentiation, but the physiological functions of many miRNAs remain to be elucidated [37]. Through bioinformatics analysis, we found that the highly conserved seed sequences of miR-183 family members exhibit high similarity, suggesting they may possess similar biological functions. In vertebrates, this family is expressed in ciliated sensory epithelial cells, cranial ganglia, and spiral ganglia [38,39]. In zebrafish, these miRNAs have been detected in ocular and nasal epithelial cells, ear sensory hair cells, and neurons [40,41], and are specifically highly expressed in organs such as the eye, nose, and inner ear [42]. A recent study on miR-182 and miR-183 found that these two miRNAs are crucial for the maturation and function of cone photoreceptors [43]. Dysfunction of miR-96 leads to the death of inner ear hair cells, resulting in progressive hearing loss in zebrafish, mice, and humans [44]. In recent years, scientists have discovered new functions of the miR-183 family in the cardiovascular system. Previously study indicated that miR-96 and miR-183 act as regulators of angiogenesis, and miR-96/miR-183 double-knockout promotes peripheral angiogenesis after myocardial infarction in mice. miR-96/miR-183 is considered potential therapeutic targets for cardiovascular diseases [29]. Exosomes derived from bone marrow mesenchymal stem cells target *FOXO1* via miR-183 to reduce apoptosis and oxidative stress in ischemia/reperfused cardiomyocytes, thereby improving cardiac function and preventing myocardial ischemia/reperfusion (MI/R) injury [45]. Additionally, miR-96 promotes myocardial infarction-induced cardiac fibrosis through the *Smad7*/*Smad3* pathway [46]. Furthermore, studies have found that miR-182 is associated with the fate determination of human endodermal cells, and miR-182 is a candidate miRNA regulator associated with the early specialization of small intestine lineage in humans [47]. However, the mechanism by which the miR-183 family influences the fate of endodermal cells and cardiac function remains unclear. In our study, both individual and combined overexpression of miR-183, miR-96, and miR-182 affected the convergence movement of endodermal cells and the migration of the mesodermal cardiac precursor cells, leading to endoderm holes and a “double heart” phenotype. These results confirm that members of the miR-183 cluster play a conserved and critical role in regulating the early embryonic endoderm-heart developmental axis.

The heart is the first organ to form and work during the development of a vertebrate. In vertebrates, bilateral precursor cell populations from the anterior mesoderm develop into the heart tube [48]. Before gastrulation, these cells respond to signals from dorsal mesoderm and endoderm cells, and are induced to become cardiac lineage cells [49]. During the somitogenesis stage, the myocardial precursor cells located between the endoderm and the extraembryonic YSL migrate towards the midline, then fuse to form a single tube and differentiate into myocardial cells. Subsequently, the heart tube bends to the right, transforming the anterior–posterior pattern into left-right asymmetry. As development progresses, the heart tube morphology undergoes cell movement, proliferation, and differentiation to form a functional heart tube [50]. These processes depend on precisely regulating multiple core transcription factors and signaling molecules. Nkx2-5 is an NK-2 homologous domain protein and one of the earliest cardiac-specific markers. In humans, mutations in NKX2-5 are associated with specific types of congenital heart defects [51]. Mutations in nkx2-5 in vertebrate embryos prevent the formation of the heart [52]. In zebrafish, the myosin heavy chain (MHC) is divided into atrial (*amhc*) and ventricular (*vmhc*) subtypes. *Vmhc* is expressed in the medial subpopulation of cardiac precursor cells in the lateral plate mesoderm and is the earliest known marker of cardiac precursor cell differentiation. *nppa* encodes atrial natriuretic peptide (ANP), which is secreted by the heart’s atria and plays a key role in the functional differentiation of cardiac muscle cells during heart development [53]. Mutations in the *nppa* gene in zebrafish lead to cardiac hypertrophy and cardiac fibrosis [54]. Bone morphogenetic protein 4 (*BMP4*) secreted by the ectoderm, is an intercellular signaling molecule responsible for the fate spectrum of adjacent mesodermal cardiac precursor cells, playing a crucial role in inducing cardiac mesoderm migration and cardiac development. Embryos with *bmp4* defects show a reduction or complete loss of mesodermal tissue [55]. The GATA family of zinc finger transcription factors is a key driver of gene regulatory networks in many organ systems during embryonic development. GATA4/5 guides mesodermal development and regulates the development of cardiac mesodermal and endodermal-derived organs [56]. *Gata4* deletion in mice leads to defects in cardiomyocyte proliferation and right ventricular morphogenesis [57]. Critically, our study demonstrates that miR-183 family overexpression significantly suppresses the expression of genes associated with early cardiac development (*p* < 0.05), resulting in abnormalities in the development and migration of myocardial precursor cells.

The endoderm plays a critical role in cardiogenesis, and its morphogenetic defects cause cardia bifida [58]. Studies involving cell transplantation demonstrate that wild-type endodermal cells rescue cardiomyocyte migration defects in *casanova*/*sox32* mutants. *sox32* is essential for early endoderm formation in zebrafish [59], and is the earliest marker gene of the endoderm. *sox17* is a downstream target of *sox32* and is related to the development of Kupffer vesicles. Together, *sox32* and *sox17* establish the molecular basis for the endoderm development and functional differentiation in left-right asymmetry in vertebrates. This study revealed that overexpression of miR-183 family members significantly suppresses the expression of the *sox32* and *sox17* genes (*p* < 0.01), leading to abnormal morphogenesis of the endoderm (formation of holes). Endoderm cells are unable to converge normally, which in turn prevents cardiac precursor cells from migrating to the midline and fusing into a single heart tube, ultimately resulting in the formation of two independent hearts.

The silencing mechanism of miRNAs is mediated by their highly conserved seed sequences of miRNAs binding to the 3’-UTR of target mRNAs through base pairing, and primarily leads to target mRNA cleavage, deadenylation, or translational inhibition [60]. We predicted through bioinformatics analysis that *s1pr2* is a potential target gene of the miR-183 family. Studies show that point mutations in the *mil* (encoding S1pr2) impair the migration of cardiac precursor cells to the midline, thereby causing cardia bifida and endodermal holes [10]. We found that the phenotype of the *s1pr2* mutation was highly consistent with that caused by the overexpression of the miR-183 family. Therefore, we speculated that there was a genetic interaction between the miR-183 family and *s1pr2*. Overexpression of miR-183 significantly suppressed the expression of the *s1pr2* gene (*p* < 0.001). To verify the direct regulatory relationship between miR-183 and *s1pr2*, we, respectively, constructed dual-luciferase reporter vectors containing the wild type (WT) and mutant type (MUT) of *s1pr2*-3’-UTR, which we then co-transfected with miRNA-NC or miR-183 mimics. Dual luciferase assay results showed that miR-183 mimic specifically inhibited the reporter activity of *s1pr2*-WT (*p* < 0.01), whereas the inhibitory effect was significantly reduced in the *s1pr2*-MUT, confirming that miR-183 regulates *s1pr2* gene expression by directly binding to its 3’-UTR. Taken together, these results support the important role of the miR-183/*s1pr2* regulatory axis in cardiac precursor cell migration and cardiac morphogenesis.

## 5. Conclusions

In summary, this study elucidates how the miR-183 family regulates endoderm convergence movements via the *s1pr2*-mediated signaling pathway, thereby influencing the directed migration of cardiac precursor cells and cardiac morphogenesis. These findings provide novel theoretical insights into the molecular mechanisms underlying congenital heart defects, such as cardia bifida, and offer important evidence for understanding the specific regulatory roles of miRNAs in cell lineage and organ development.

## Data Availability

The results presented in this study are included in the article. Further inquiries can be directed to the corresponding author.

## Supplementary Materials

The following supporting information can be downloaded at https://www.mdpi.com/article/10.3390/biom15101434/s1: Table S1: Primer used to amplify template DNA to synthesize RNA probes of WISH; Table S2: Primer used for real-time quantitative PCR.
